# Supplementary material for: An interpretable bimodal neural network characterizes the sequence and preexisting chromatin predictors of induced transcription factor binding
Source: Genome Biol. 2021 Jan 7;22:20. doi: 10.1186/s13059-020-02218-6 (PMC7788824; doi:10.1186/s13059-020-02218-6)
Supplement: Supplementary file 1 — Additional file 1. Integrated supplementary figures. Contains supplementary figures from S1 to S12. [file 13059_2020_2218_MOESM1_ESM.docx]

**Supplementary Figures**


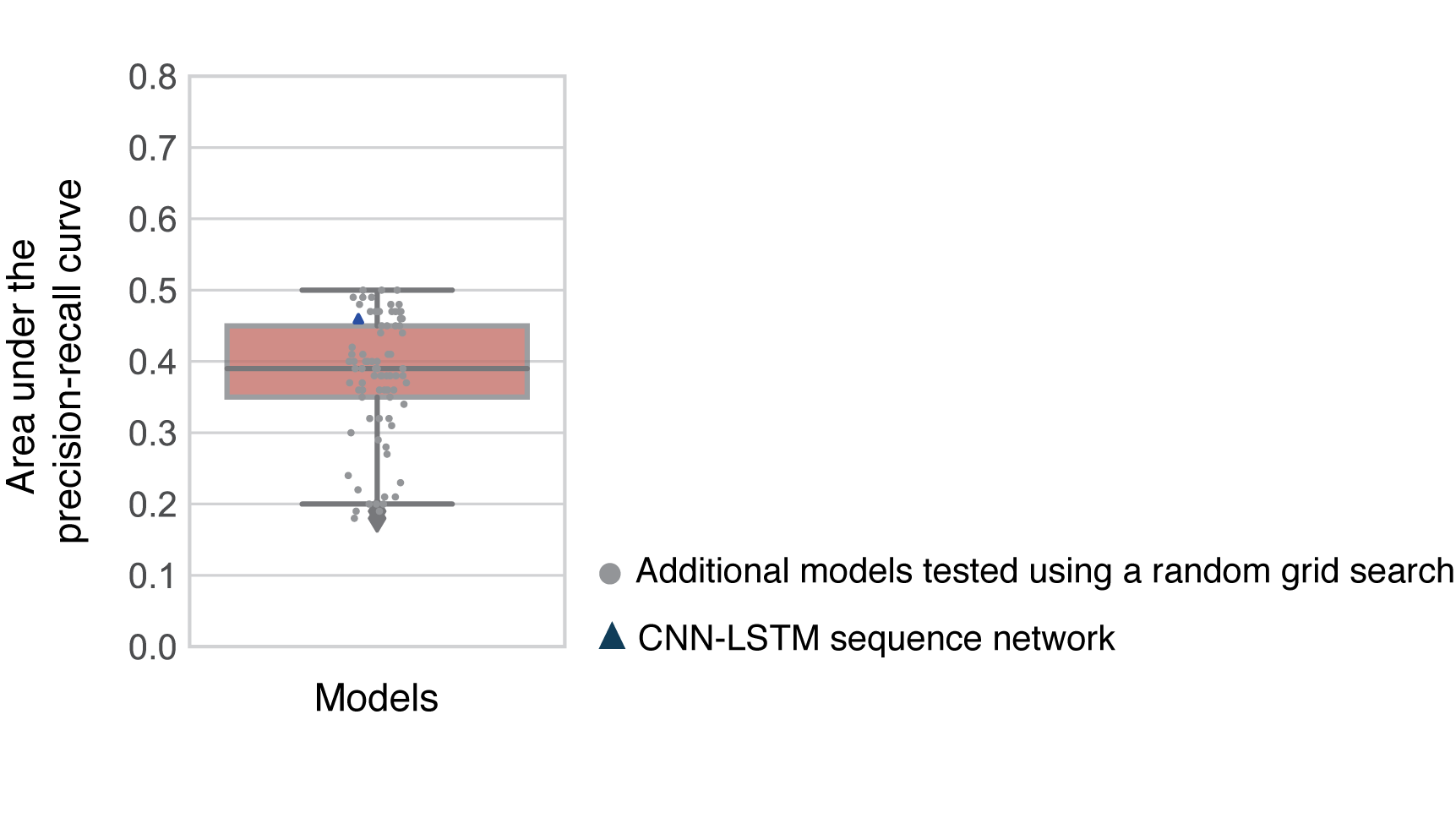


**Figure S1: Performance comparison between the bimodal CNN-LSTM architecture employed by Bichrom and bimodal CNN architectures with varying hyper-parameters and numbers of layers.** Performance is assessed as area under the precision-recall curve when predicting Ascl1 binding (mEB+12hrs) using sequence and preexisting mEB and mES chromatin features (chr10 held-out test set, chr17 validation). The alternate CNN architectures were chosen using a random grid search over the following hyper-parameters: (1) number of convolution layers, (2) convolutional kernel size, (3) number of convolutional filters, (4) max pooling size, (5) max pooling stride, (6) number of dense layers, (7) number of dense nodes and (8) dropout rate to select network hyper-parameters (search values shown in Table S7).

**
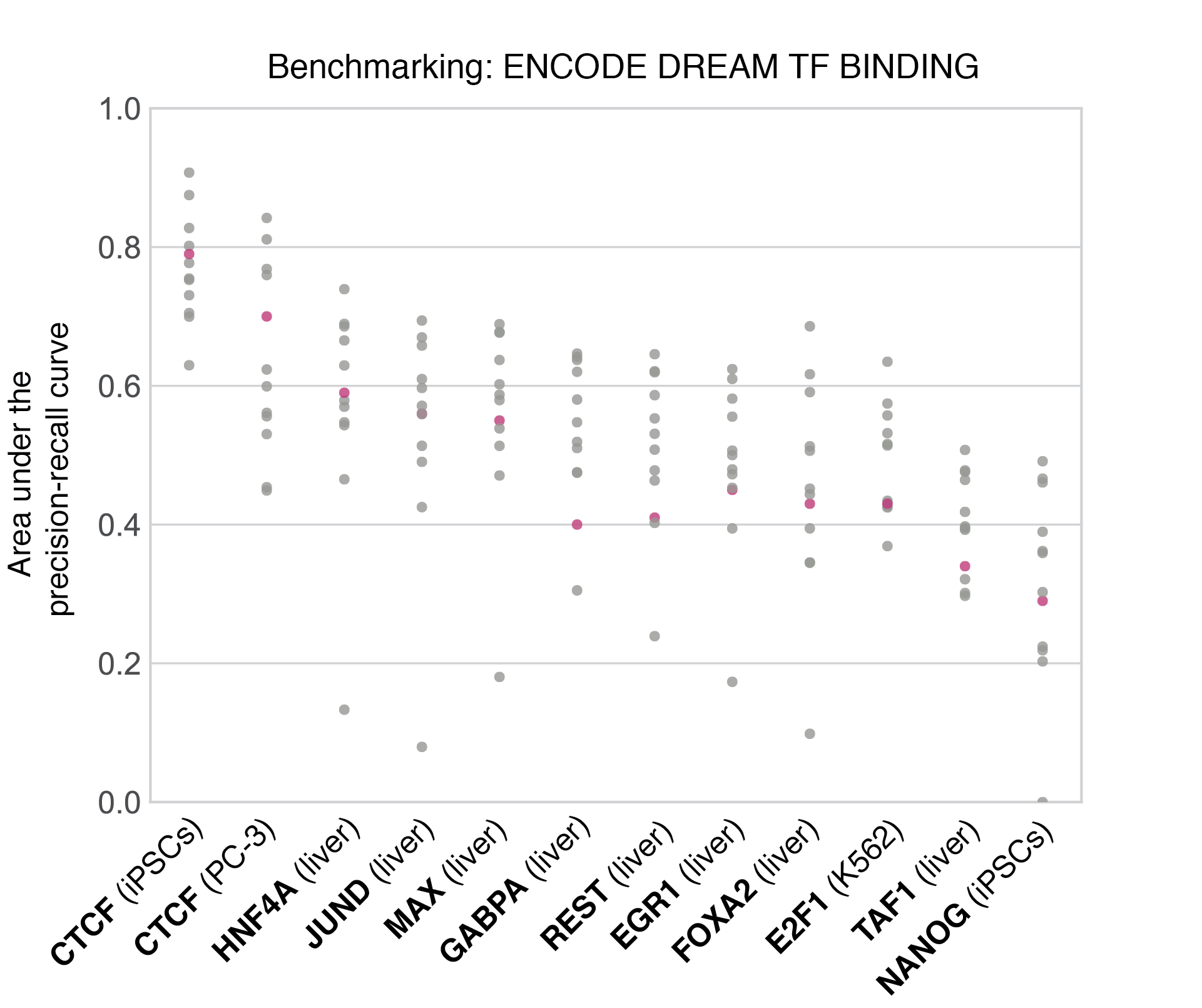
**

**Figure S2: Performance comparisons between Bichrom and alternative TF binding prediction models.** Plot shows area under the precision-recall curve for the Bichrom architecture (red) run using sequence and concurrent chromatin accessibility (DNase-seq) as inputs for predicting within-cell-type genome-wide TF binding, compared to auPRCs of the top 10 methods from the ENCODE-DREAM TF binding prediction challenge.


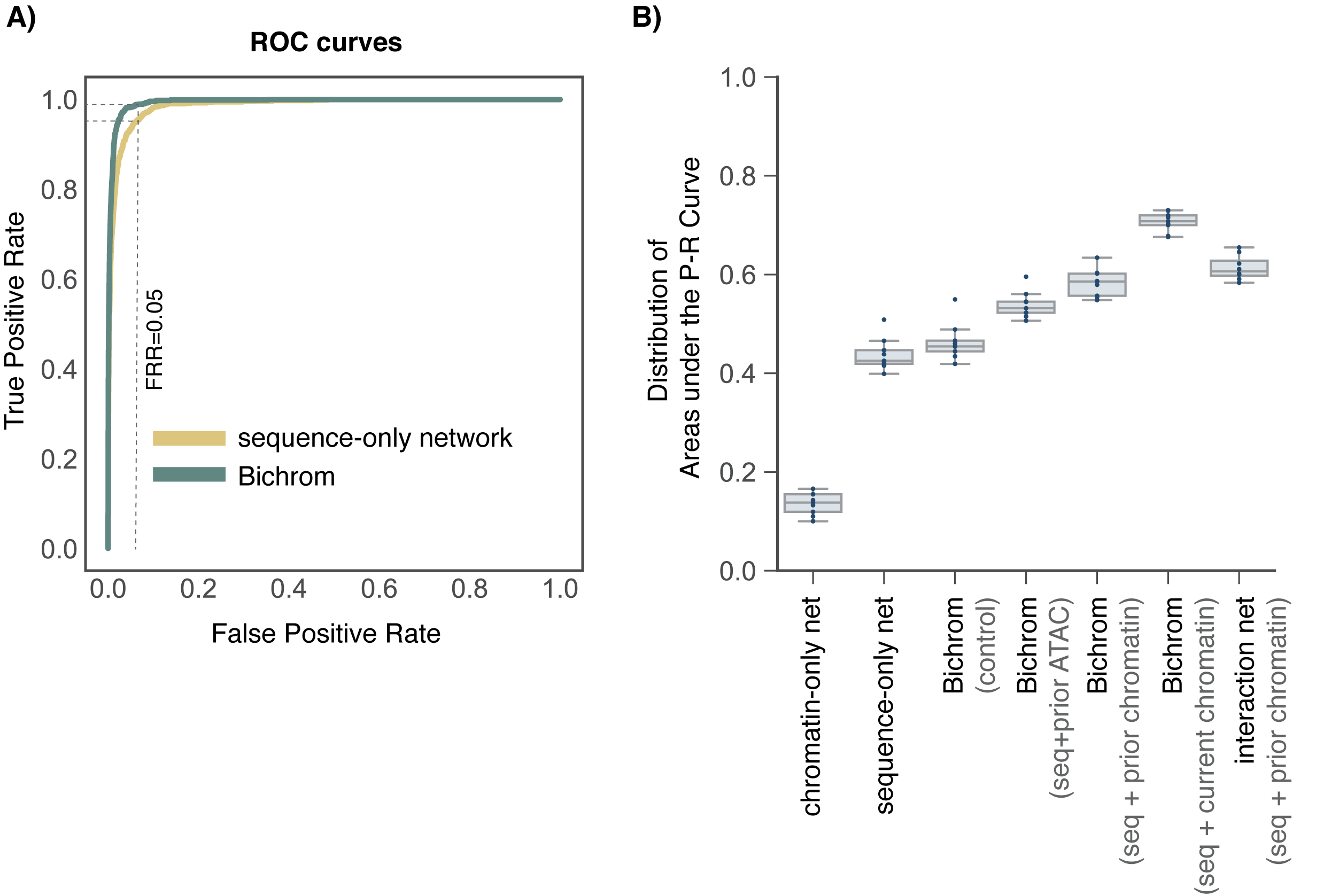
­­

**Figure S3: Assessment of the relative increase in Bichrom performance due to incorporation of preexisting chromatin features. A)** The receiver-operating characteristic (ROC) curves for the sequence-only network and Bichrom trained to predict Ascl1 binding. At a fixed false positive rate (FPR) of 0.05, both models have high true positive rates (TPR > 0.9). **B)** Model auPRC distributions for various neural networks over 9 independent training sets (predicting Ascl1 binding). The performance of Bichrom’s additive bimodal network architecture is comparable to that of an early integration network (interaction net), confirming that an additive model does not result in a loss of predictive accuracy.

**
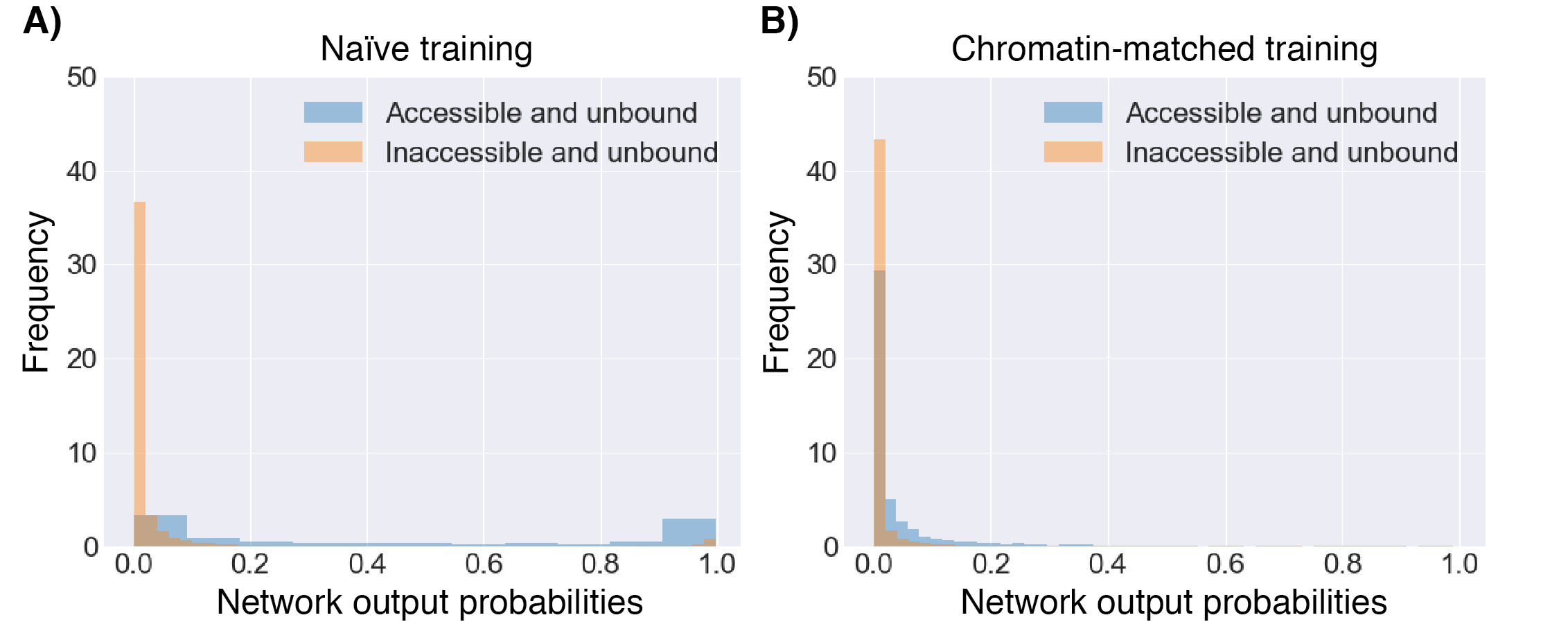
­**

**Figure S4: The distribution of Bichrom_SEQ_ sub-network scores at Ascl1-unbound sites divided according to preexisting accessibility status.** **A)** In the naïve training, a large number of pre-accessible unbound regions (blue) are incorrectly assigned high Bichrom_SEQ_ scores. **B)** This bias is lost in the chromatin-matched training approach, with the model behaving more uniformly across pre-accessible and pre-inaccessible sites.


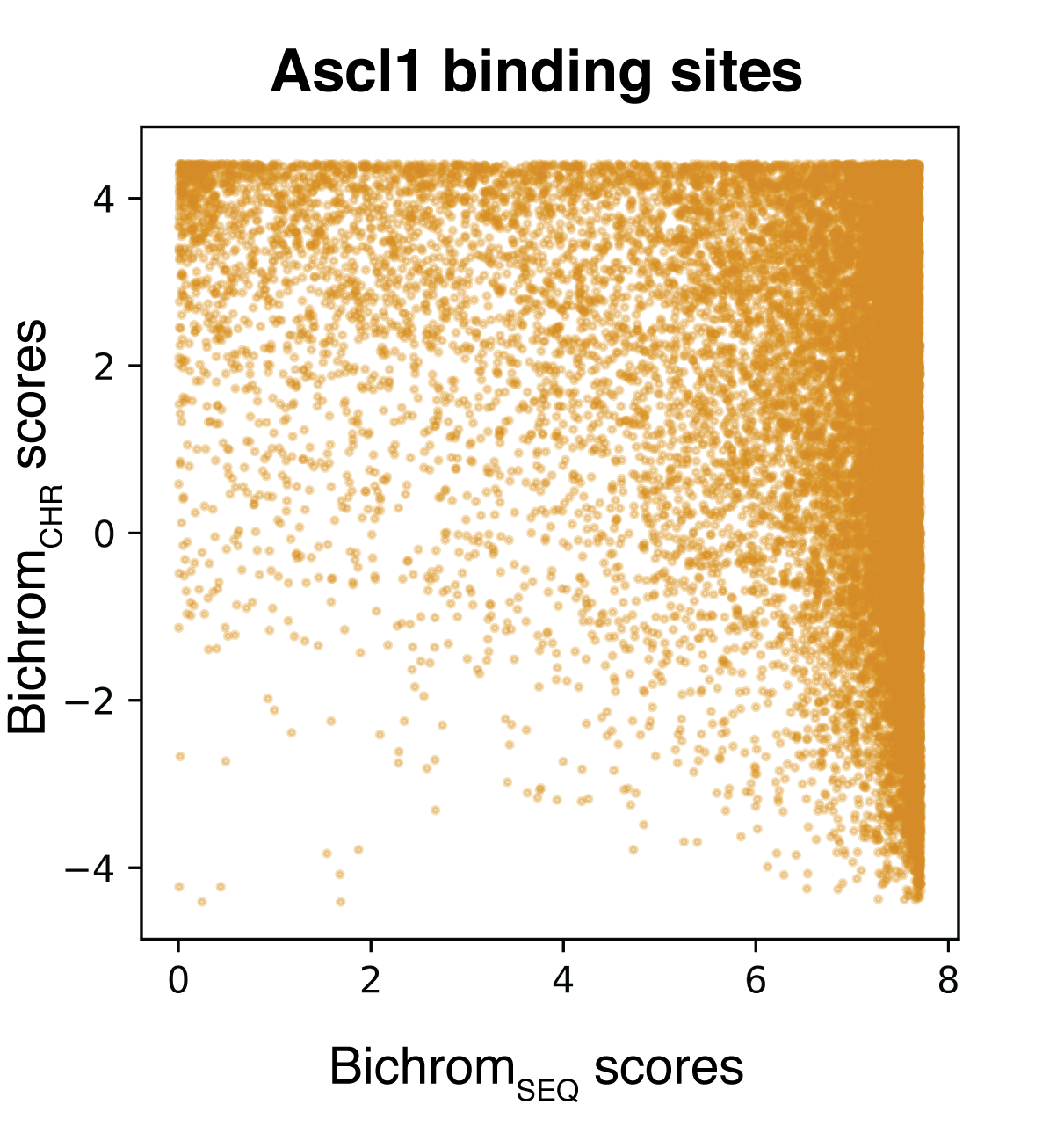


**Figure S5: Network parameterization is not responsible for the observed sequence-chromatin compensation.** Sequence and prior chromatin sub-network embeddings for Ascl1 from a control bimodal neural network in which all sequence sub-network weights are kept fixed (non-trainable) while training the network on genome-wide binding data (bound genomic sites as positive training examples and a larger set of randomly selected unbound sites are negative training examples).

**
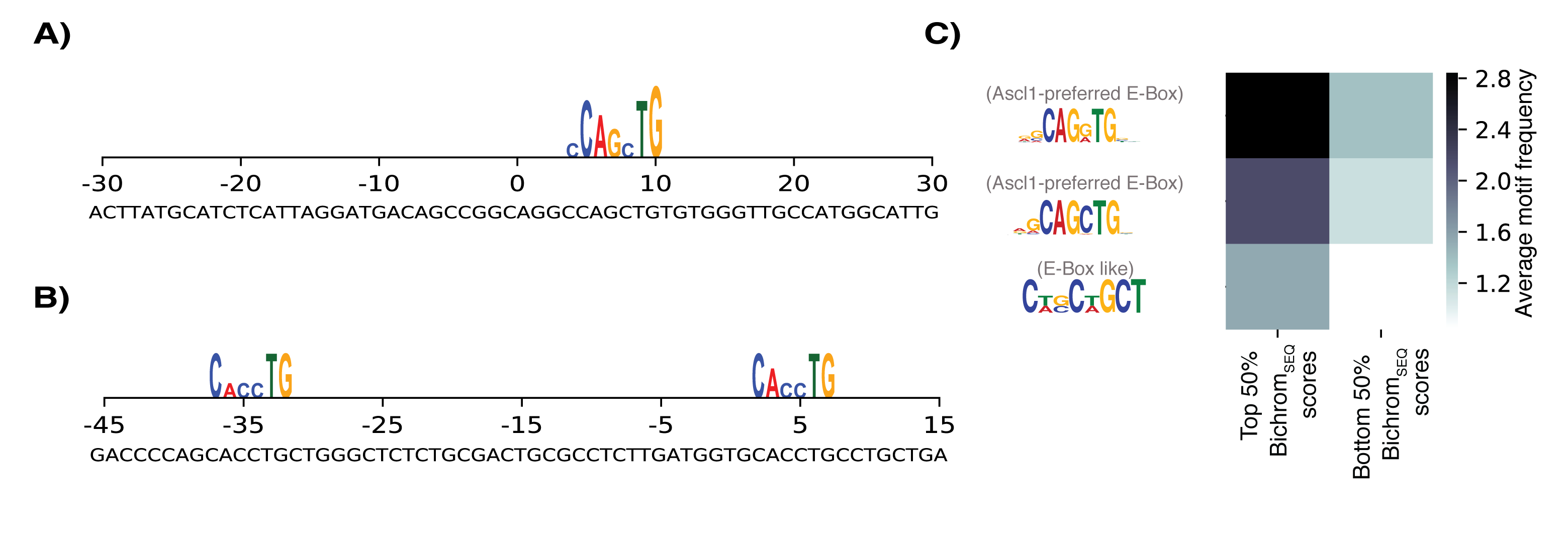
**

**Figure S6: Bichrom learns Ascl1’s cognate binding motif as the primary sequence feature of Ascl1 binding sites. A)** & **B)** Feature attribution with integrated gradients at two sample Ascl1 binding sites (chr10:4710120-4710170 and chr10:28136730-28136800). **C)** MEME-ChIP-determined E-box motif frequency at Ascl1 binding sites assigned the bottom 50% Bichrom_SEQ_ sub-network scores compared to sites assigned the top 50% Bichrom_SEQ_ sub-network scores.


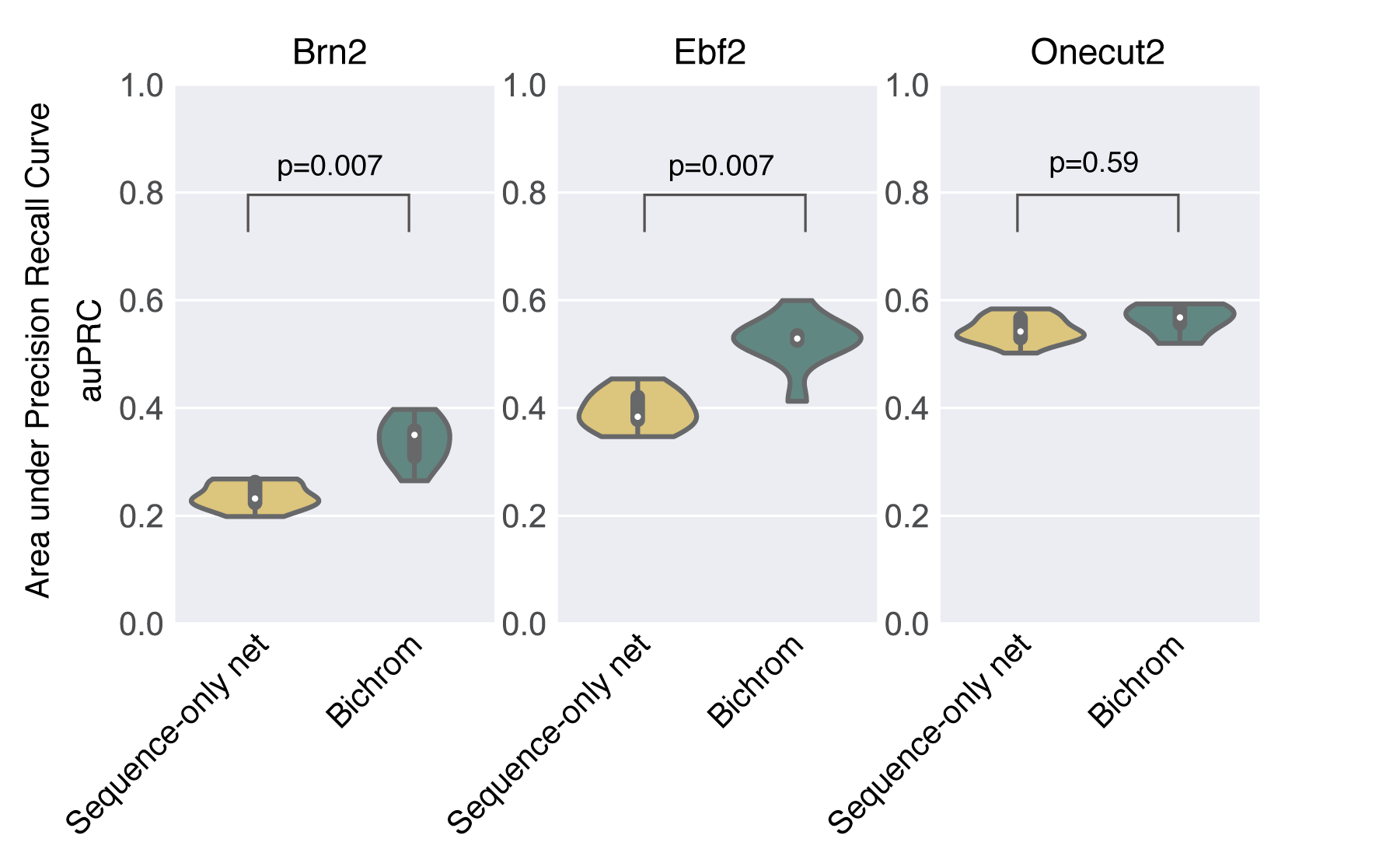


**Figure S7: Brn2, Ebf2, and Onecut 2 vary in their relative dependence on preexisting chromatin features.** Plot shows auPRC distributions for sequence-only and Bichrom networks trained on 9 independent training sets, each comprising of a distinct chromosome as a held-out test set for Brn2, Ebf2 and Onecut2.


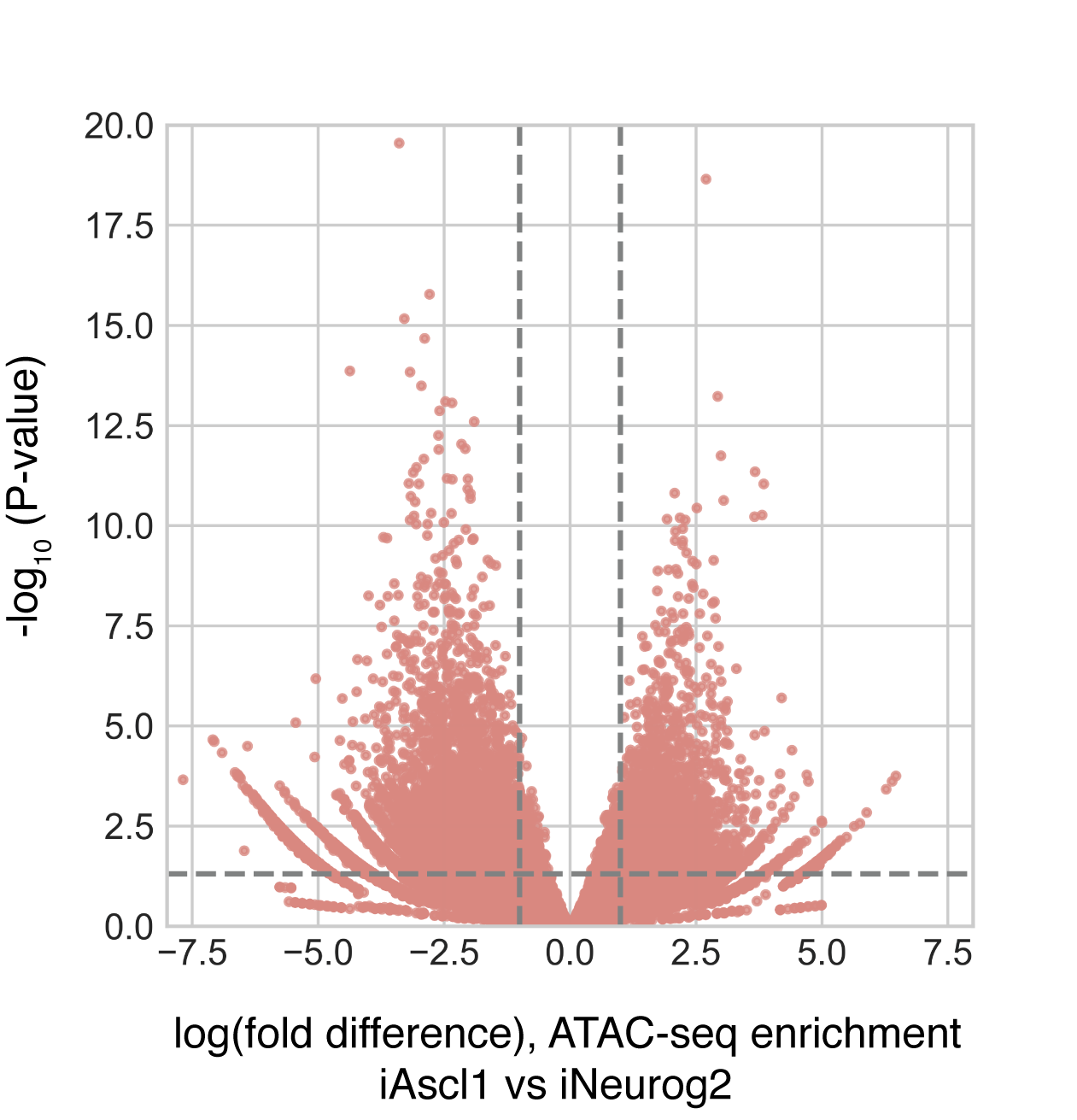


**Figure S8: Ascl1 and Neurog2 induce distinct accessibility landscapes when induced in mEB cells.** Differences in chromatin accessibility (ATAC-seq) levels in iAscl1+12hrs compared to iNeurog2+12hrs, as assessed using DESEQ2. The volcano plot maps the log fold-change and p-values at the union of accessible domains in iAscl1 and iNeurog2 neurons.

**
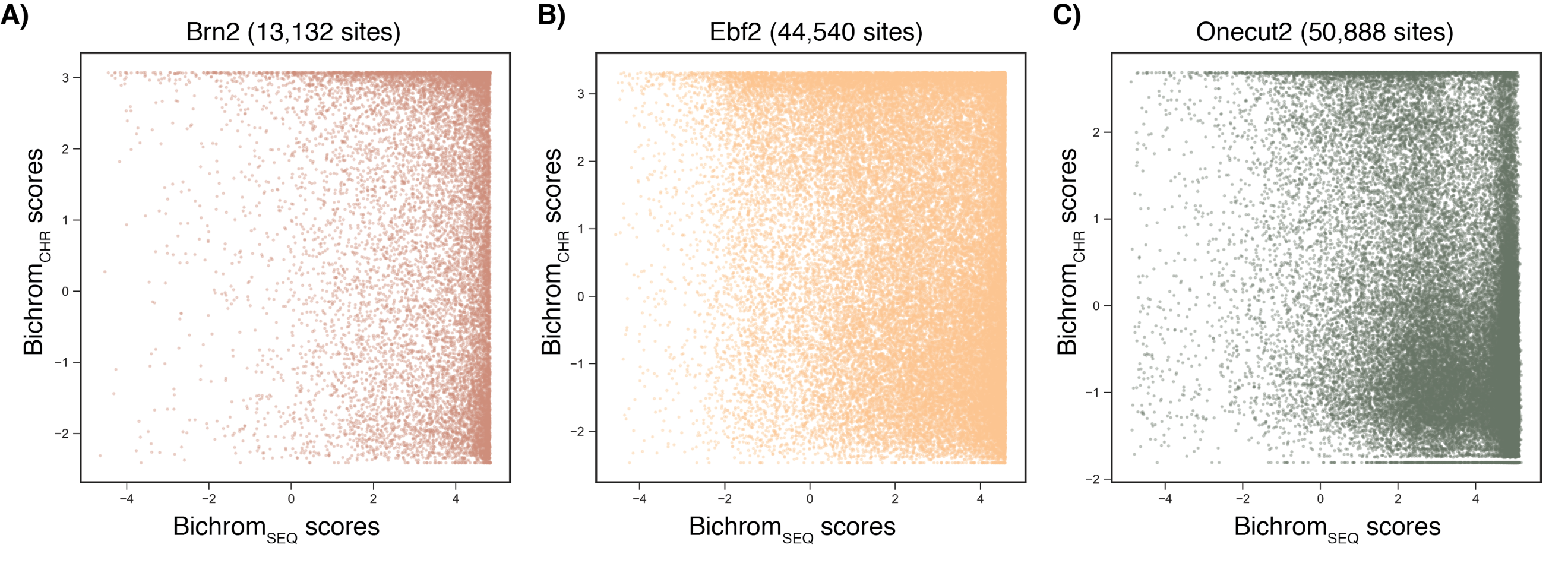
**

**Figure S9: Bichrom-derived latent embeddings for neuronal TFs.** **A)** Brn2, **B)** Ebf2 and **C)** Onecut2 induced in EB-derived iAscl1 cells.


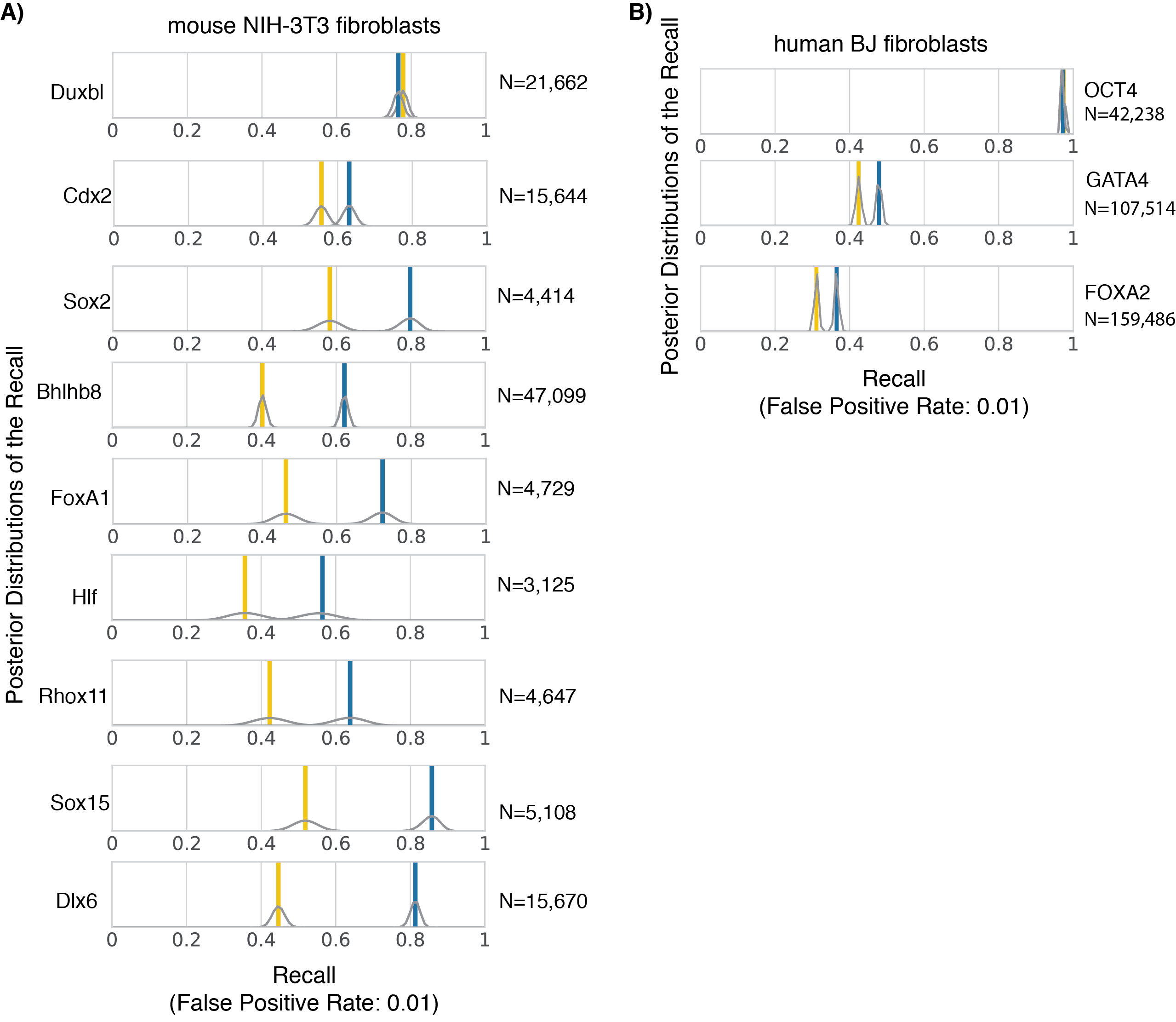


**Figure S10: Induced TFs vary in their relative dependence on preexisting chromatin features.** Bichrom recall at a fixed false positive rate (0.01) for **A)** the 9 mouse NIH-3T3 induced TFs and **B)** the 3 human fibroblast induced TFs. Chromosome 17 is used as a validation set, chromosome 11 is used as a held-out test set. The posterior distributions of the recall (estimated using a Beta uniform prior) are also plotted.


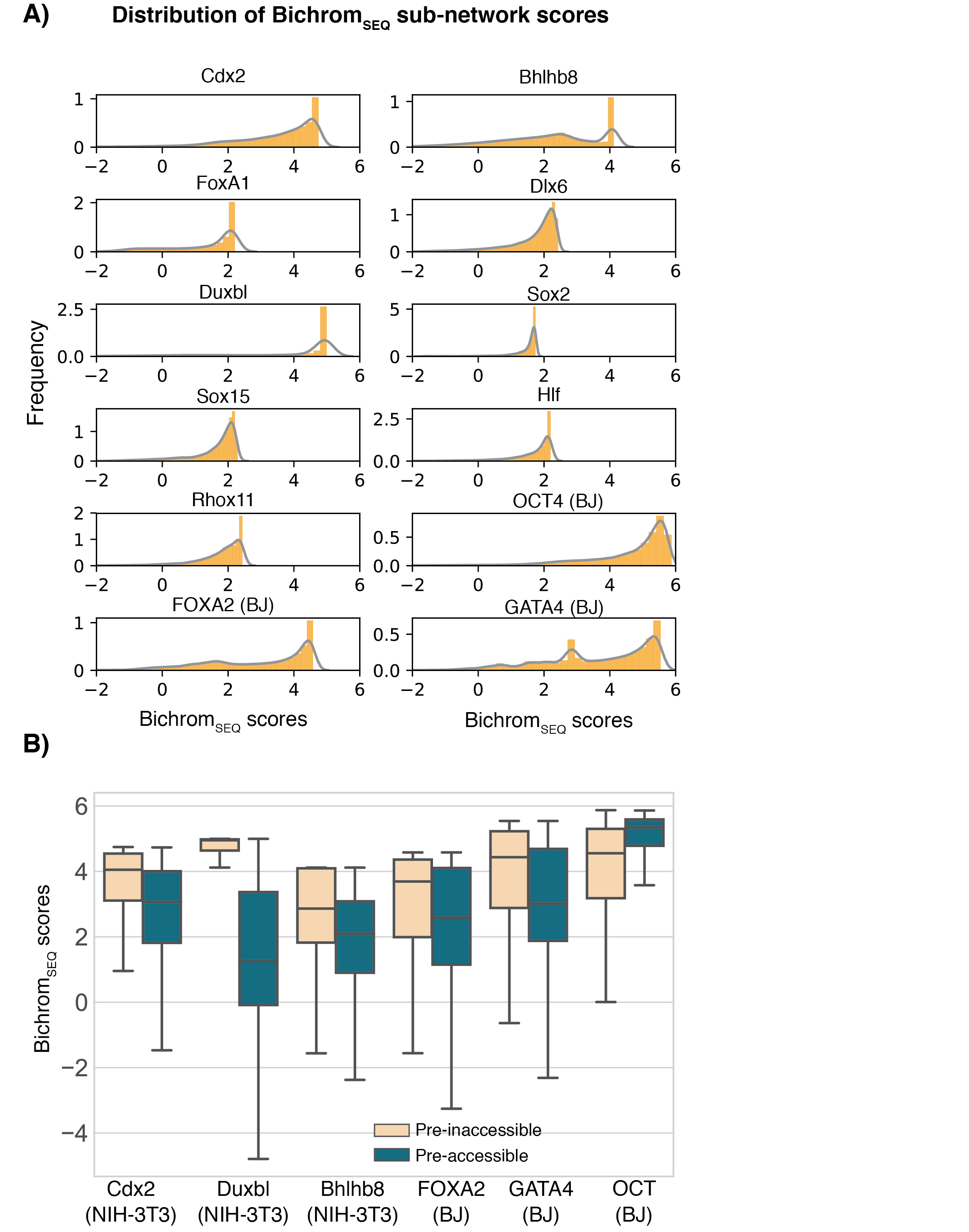


**Figure S11: Induced TFs typically display stronger sequence motif features at pre-inaccessible binding sites. A)** The distribution of Bichrom_SEQ_ sub-network scores for 9 TFs induced in mouse NIH-3T3 fibroblasts and 3 TFs induced in human BJ fibroblasts. **B)** The distributions of Bichrom_SEQ_ sub-network scores at pre-accessible versus pre-inaccessible binding sites are plotted for Cdx2, Duxbl, Bhlhb8, FOXA2, GATA4 and OCT4.


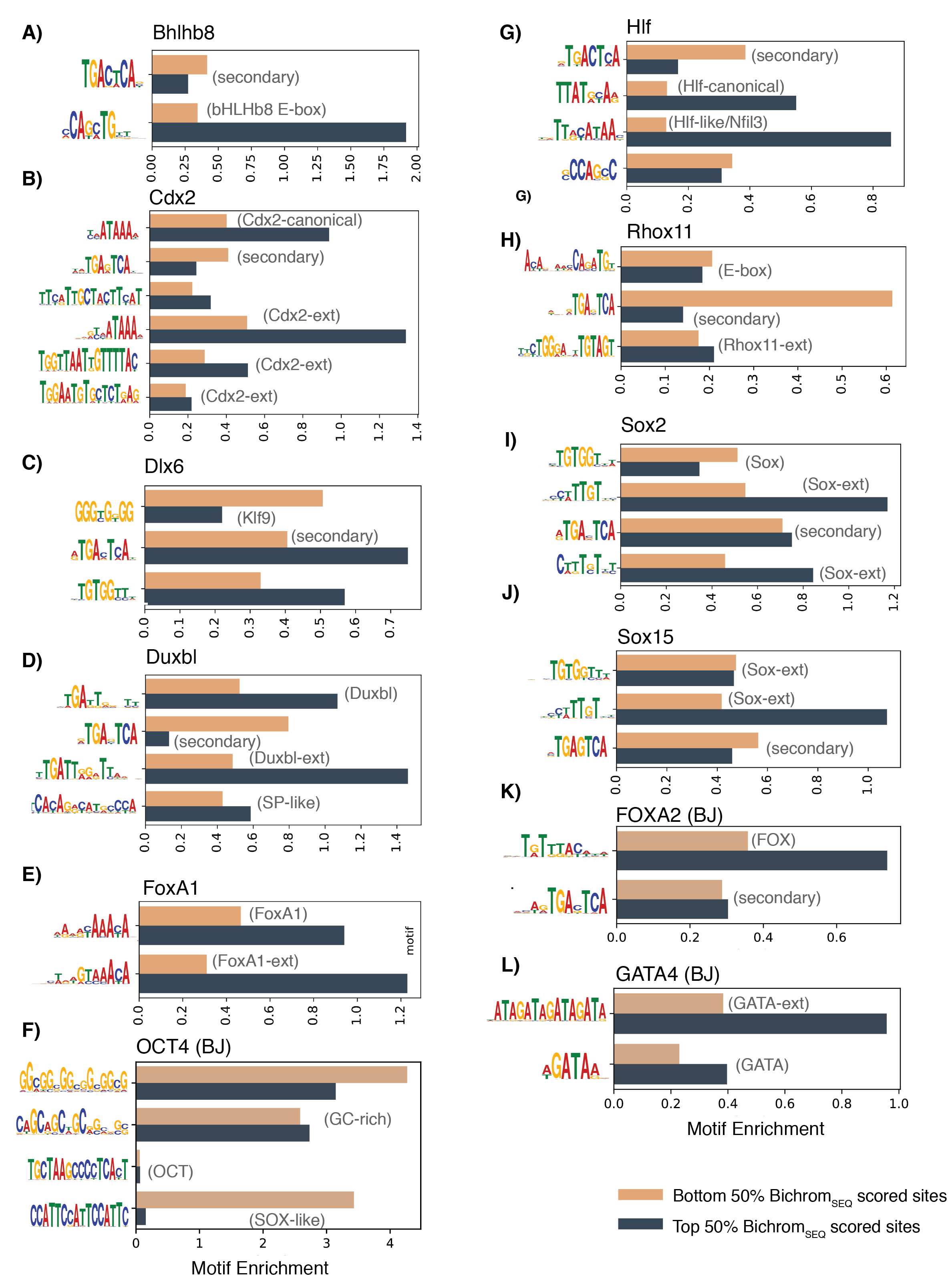


**­**

**Figure S12: Analysis of MEME-ChIP-determined motifs for each induced TF.** Individual plots show motif enrichment at sites assigned the bottom 50% Bichrom_SEQ_ sub-network scores compared to motif enrichment at sites assigned the top 50% Bichrom_SEQ_ sub-network scores for **A)** bHLHb8, **B)** Cdx2, **C)** Dlx6, **D)** Duxbl, **E)** FoxA1, **F)** OCT4 (BJ), **G)** Hlf, **H)** Rhox11, **I)** Sox2, **J)** Sox15, **K)** FOXA2 (BJ) and **L)** GATA4 (BJ).
