# Supplementary material for: An interpretable bimodal neural network characterizes the sequence and preexisting chromatin predictors of induced transcription factor binding
Source: Genome Biol. 2021 Jan 7;22:20. doi: 10.1186/s13059-020-02218-6 (PMC7788824; doi:10.1186/s13059-020-02218-6)
Supplement: Supplementary file 2 — Additional file 2. Supplemental data and data source descriptions. Contains supplementary tables from S1 to S7 and all data source citations. [file 13059_2020_2218_MOESM2_ESM.docx]

**Supplemental Data and Data Source Descriptions**

**Table S1:** Prior chromatin datasets for mouse embryoid bodies, generated in the same cell line as that were used to induce Ascl1.

| **Data** | **Replicates** | **Cell Type** | **Cell Line** |
| --- | --- | --- | --- |
| ATAC-seq^1^ | 2 | EB | Ainv15 |
| H3K27ac^1^ | 2 | EB | Ainv15 |
| H3K27me3^1^ | 2 | EB | Ainv15 |
| H3K4me1^1^ | 2 | EB | Ainv15 |
| H3K4me2^1^ | 2 | EB | Ainv15 |
| H3K4me3^1^ | 2 | EB | Ainv15 |

**Table S2:** Prior chromatin datasets from mouse embryonic stem cells, sourced from the referenced publications.

| **Data** | **Replicates** | **Cell Type** | **Cell Line** |
| --- | --- | --- | --- |
| H3K9ac^2^ | 2 | ES | E14 |
| H3K9me3^2^ | 2 | ES | E14 |
| H3K36me3^2^ | 2 | ES | E14 |
| H2A.Z^3^ | 1 | ES | V6.5 |
| acH2A.Z^3^ | 1 | ES | V6.5 |
| H4K20me3^4^ | 2 | ES | V6.5 |

**Table S3:** ChIP-seq data for TFs induced in NIH3T3 fibroblasts, downloaded from Raccaud *et al*^5^, mEB induced ChIP-seq data downloaded from Aydin *et al.* ^6^ and human BJ induced data, downloaded from Donaghey *et al*^7^*.* Processed with bowtie (1.0.1) and multiGPS (version 0.74).

| **Data** | **Cell Type** | **Number of Peaks** | **Replicates** | **multiGPS Signal Fraction** |
| --- | --- | --- | --- | --- |
| bHLHb8 | NIH-3T3 | 47,106 | 2 | 0.083,0.117 |
| Cdx2 | NIH-3T3 | 15,650 | 1 | 0.029 |
| Dlx6 | NIH-3T3 | 15,163 | 1 | 0.027 |
| Duxbl | NIH-3T3 | 21,668 | 1 | 0.04 |
| FoxA1 | NIH-3T3 | 4,736 | 2 | 0.014,0.010 |
| Hlf | NIH-3T3 | 3,131 | 1 | 0.009 |
| Rhox11 | NIH-3T3 | 4,653 | 1 | 0.01 |
| Sox15 | NIH-3T3 | 5,114 | 1 | 0.013 |
| Sox2 | NIH-3T3 | 4,421 | 1 | 0.019 |
| Neurog2 | mES | 26,643 | 3 | 0.24, 0.11, 0.18 |
| Ascl1 | mES | 21,176 | 3 | 0.08, 0.11, 0.12 |
| OCT4 | BJ | 159,485 | 2 | 0.29, 0.29 |
| FOXA2 | BJ | 42,37 | 1 | 0.1 |
| GATA4 | BJ | 107,513 | 1 | 0.22 |

**Table S4:** ATAC-seq experiments used as prior chromatin data in mouse NIH3T3 fibroblasts^5^.

| **Data** | **Replicates** | **Cell Type** | **Cell Line** |
| --- | --- | --- | --- |
| ATAC-seq | 2 | Mouse Fibroblasts | NIH3T3 |

**Table S5**: Experiments used as prior chromatin data in human BJ fibroblasts^7^.

| **Data** | **Replicates** | **Cell Type** | **Cell Line** |
| --- | --- | --- | --- |
| ATAC-seq | 2 | Human Fibroblasts | BJ |
| H2K27ac | 2 | Human Fibroblasts | BJ |
| H3K4me2 | 2 | Human Fibroblasts | BJ |
| H3K27me3 | 2 | Human Fibroblasts | BJ |

**Table S6:** Bichrom network parameters tested within the hyper-parameter random grid search.

| **Parameter** | **Options** |
| --- | --- |
| Number of convolutional filters | 16, 32, 64, 128, 256 |
| Number of dense layers | 2-5 |
| Size of dense layers | 128, 512, 1024, 2048 |
| Activation functions | tanh, relu, sigmoid |
| Dropout Rate | 0.5, 0.25 |

**Table S7:** Network parameters used to construct alternative CNN-based networks (which are compared with the CNN-LSTM in Fig. S1)

| **Parameter** | **Options** |
| --- | --- |
| Number of convolutional filters | 16, 32, 64, 128, 256 |
| Number of dense layers | 1-10 |
| Size of dense layers | 128, 512, 1024, 2048 |
| Activation functions | tanh, relu, sigmoid |
| Dropout Rate | 0.5, 0.25 |
| Size of convolutional filters | 6, 15, 24, 32 |
| Max pooling size | 2, 4, 6, 8, 10, 15 |
| Max pooling stride | 2, 4, 6, 8, 10, 15 |
| Number of convolutional layers | 3-10 |

**Data Sources**

1. Velasco, Silvia, Mahmoud M. Ibrahim, Akshay Kakumanu, Görkem Garipler, Begüm Aydin, Mohamed Ahmed Al-Sayegh, Antje Hirsekorn, et al. “A Multi-Step Transcriptional and Chromatin State Cascade Underlies Motor Neuron Programming from Embryonic Stem Cells.” *Cell Stem Cell* 20 (2): 205–217.e8. **GSE80483**. Gene Expression Omnibus. <https://doi.org/10.1016/j.stem.2016.11.006>. (2017)
2. Yue, Feng, Yong Cheng, Alessandra Breschi, Jeff Vierstra, Weisheng Wu, Tyrone Ryba, Richard Sandstrom, et al. “A Comparative Encyclopedia of DNA Elements in the Mouse Genome.” *Nature* 515 (7527). Nature Publishing Group: 355–64. **GSE49847**. Gene Expression Omnibus. <https://doi.org/10.1038/nature13992>. (2014)
3. Ku, Manching, Jacob D Jaffe, Richard P Koche, Esther Rheinbay, Mitsuhiro Endoh, Haruhiko Koseki, Steven A Carr, and Bradley E Bernstein. “H2A.Z Landscapes and Dual Modifications in Pluripotent and Multipotent Stem Cells Underlie Complex Genome Regulatory Functions.” *Genome Biology* 13 (10): R85. **GSE39237**. Gene Expression Omnibus. <https://doi.org/10.1186/gb-2012-13-10-r85>. (2012)
4. Mikkelsen, Tarjei S., Manching Ku, David B. Jaffe, Biju Issac, Erez Lieberman, Georgia Giannoukos, Pablo Alvarez, William Brockman, Tae-Kyung Kim, Richard P. Koche, William Lee, Eric Mendenhall, Aisling O’Donovan, Aviva Presser, Carsten Russ, Xiaohui Xie, Alexander Meissner, Marius Wernig, Rudolf Jaenisch, Chad Nusbaum, Eric S. Lander & Bradley E. Bernstein. “Genome-Wide Maps of Chromatin State in Pluripotent and Lineage-Committed Cells.” *Nature* 448 (7153). Nature Publishing Group: 553–60. **GSE12241**. Gene Expression Omnibus. <https://doi.org/10.1038/nature06008>. (2007)
5. Raccaud, Mahé, Elias T Friman, Andrea B Alber, Harsha Agarwal, Cédric Deluz, Timo Kuhn, J Christof M Gebhardt, and David M Suter. “Mitotic Chromosome Binding Predicts Transcription Factor Properties in Interphase.” *Nature Communications* 10 (1): 487. **GSE119784**. Gene Expression Omnibus. <https://doi.org/10.1038/s41467-019-08417-5>. (2019)
6. Aydin, Begüm, Akshay Kakumanu, Mary Rossillo, Mireia Moreno-Estellés, Görkem Garipler, Niels Ringstad, Nuria Flames, Shaun Mahony, and Esteban O. Mazzoni. Proneural Factors Ascl1 and Neurog2 Contribute to Neuronal Subtype Identities by Establishing Distinct Chromatin Landscapes. *Nature Neuroscience* 22 (6): 897–908. **GSE114176**. Gene Expression Omnibus. <https://doi.org/10.1038/s41593-019-0399-y>. (2019).
7. Donaghey, Julie, Sudhir Thakurela, Jocelyn Charlton, Jennifer S Chen, Zachary D Smith, Hongcang Gu, Ramona Pop, Kendell Clement, Elena K. Stamenova, Rahul Karnik, David R. Kelley, Casey A. Gifford, Davide Cacchiarelli, John L. Rinn, Andreas Gnirke, Michael J. Ziller & Alexander Meissner. “Genetic Determinants and Epigenetic Effects of Pioneer-Factor Occupancy.” *Nature Genetics* 50 (2): 250–58. **GSE90456**. Gene Expression Omnibus. <https://doi.org/10.1038/s41588-017-0034-3>. (2018)

**Supplementary Material References**

1. Velasco, S. *et al.* A Multi-step Transcriptional and Chromatin State Cascade Underlies Motor Neuron Programming from Embryonic Stem Cells. *Cell Stem Cell* **20,** 205–217.e8 (2017).

2. Yue, F. *et al.* A comparative encyclopedia of DNA elements in the mouse genome. *Nature* **515,** 355–364 (2014).

3. Ku, M. *et al.* H2A.Z landscapes and dual modifications in pluripotent and multipotent stem cells underlie complex genome regulatory functions. *Genome Biol.* **13,** R85 (2012).

4. Mikkelsen, T. S. *et al.* Genome-wide maps of chromatin state in pluripotent and lineage-committed cells. *Nature* **448,** 553–560 (2007).

5. Raccaud, M. *et al.* Mitotic chromosome binding predicts transcription factor properties in interphase. *Nat. Commun.* **10,** 487 (2019).

6. Aydin, B. *et al.* Proneural factors Ascl1 and Neurog2 contribute to neuronal subtype identities by establishing distinct chromatin landscapes. *Nat. Neurosci.* **22,** 897–908 (2019).

7. Donaghey, J. *et al.* Genetic determinants and epigenetic effects of pioneer-factor occupancy. *Nat. Genet.* **50,** 250–258 (2018).
